# Therapeutic effects of a combination of *Chinese quince* and *Saururus chinensis* extract on allergic airway inflammation in an ovalbumin-induced asthma mouse model

**DOI:** 10.3389/fnut.2025.1613413

**Published:** 2025-07-10

**Authors:** Hye Jin Lee, Ki Cheon Kim, Woo Jin Lee, Hyo Jung Nam, Su Jin Ryu, Seon Hyeok Kim, Won Jun Kim, Jae Won Yoon, Tae-Hee Lee, Pan-Young Jeong

**Affiliations:** ^1^Department of Efficacy Evaluation, Centralbio Co., Ltd., Incheon, Republic of Korea; ^2^Life Science Research Institute, Novarex Co., Ltd., Cheongju, Chungbuk, Republic of Korea

**Keywords:** allergic asthma, type 2 immune response, ovalbumin, inflammatory response, mucus production, *Chinese quince*, *Saururus chinensis*, STAT6

## Abstract

**Background:**

Allergic asthma involves chronic inflammation, airway remodeling, and hyperresponsiveness. Inhaled corticosteroids combined with long-acting β2 agonists are effective; however, some patients experience side effects, highlighting the need for safer natural alternatives suitable for long-term use. *Chinese quince* (Q) and *Saururus chinensis* (SC) are used to treat various diseases, including asthma and inflammation. Q and SC extracts contain bioactive compounds that help modulate airway inflammation. Therefore, combining the two may enhance their immunomodulatory effects. However, the effects of a Q/SC mixture on allergic asthma remain unclear. The aim of this study is to assess the therapeutic effectiveness of a Q/SC mixture in treating asthma.

**Methods:**

The therapeutic efficacy of the Q/SC extract was evaluated in an ovalbumin (OVA)-induced allergic airway inflammation model. After euthanasia, we assessed cell counts, cytokine expression in the bronchoalveolar lavage fluid (BALF), blood immunoglobulin (Ig) E levels, inflammatory cell infiltration, mucus production in the lung tissue, and the expression of protein and cytokine.

**Results:**

A high-concentration Q/SC extract significantly reduced total cell and eosinophil counts, cytokine expression in BALF, and serum IgE levels. Furthermore, it reduced the expression of type 2 cytokines (IL-4, IL-5, IL-13) and inducible nitric oxide synthase in lung tissue. The extract also attenuated inflammatory cell infiltration and mucus production while inhibiting the STAT6 signaling pathway.

**Conclusion:**

A high concentration of Q/SC extract effectively alleviates allergic airway inflammation by reducing eosinophilic inflammation, type 2 cytokine secretion, and mucus hyperproduction. This suggests that it could be a potential remedy for managing allergic airway inflammation.

## 1 Introduction

Asthma is a convoluted respiratory condition marked by heightened airway sensitivity, ongoing inflammation, and structural changes. These factors can lead to symptoms ranging from mild wheezing to severe, life-threatening obstructions (1). Importantly, despite advancements in understanding its underlying mechanisms, asthma remains a significant global health challenge (2, 3). Various allergens (air pollution and house dust mites) and viral infections can trigger asthma. The key pathological features include epithelial cell hyperplasia, mucus hypersecretion, pulmonary fibrosis, and inflammatory cell infiltration. These elements collectively contribute to disease progression and severity (4, 5). The mucus plays a vital function in the host defense of the airway; however, excessive mucus production can lead to airway obstruction and worsen various respiratory diseases. MUC5AC is important in mucus hyperproduction in asthma, with approximately 20 mucin genes involved in mucus secretion (6–8). Type 2 cytokines interleukin (IL)-4 and IL-13 induce mucus production in the airways, with IL-13 playing a significant role in excessive mucus production associated with asthma. The key transcription factor STAT6, triggered by IL-4 and IL-13 through the IL-4Rα subunit, is crucial in modulating MUC5AC gene expression (9, 10).

The inflammatory response in asthma is triggered by various inflammatory cells, including mast cells, B cells, T cells, neutrophils, eosinophils, and cytokines. T helper 2 (Th2) cells are highly involved in the development and progression of allergic asthma. Th2 cells release cytokines IL-4, IL-5, and IL-13, which stimulate immunoglobulin (Ig) E synthesis and recruit eosinophils to the site of inflammation. This process leads to excessive mucus secretion and airway inflammation (11). Therefore, Th2 cell immune regulation has been recognized as a promising therapeutic strategy for treating asthma and monoclonal antibody drugs that regulate type 2 cytokines have been approved (12). However, asthma and allergies have complex mechanisms involving many cells, making it potentially inadequate to target a single pathologic mechanism. Furthermore, the main pharmacological approach for treating asthma is the daily use of inhaled corticosteroids combined with long-acting β2 agonists. Inhaled corticosteroids combined with long-acting β2 agonists therapy achieves excellent results in most patients; nevertheless, approximately 10–25% of patients experience persistent asthma symptoms. Moreover, corticosteroid side effects, including pneumonia, hypertension, hyperlipidemia, myopathy, and cataracts, have been reported (13). Therefore, asthma treatments derived from natural products that are free of side effects and suitable for long-term use are needed.

Medicinal plant extracts may exert multiple effects rather than blocking a single cell or mechanism, increasing their potential for development as asthma treatments. *Chinese quince* (Q) is a medicinal plant species from the Rosaceae family. It has been used to treat various diseases in Japan, Korea, and China. Q extract contains bioactive compounds (phenolic and triterpene compounds) that are known to have antibacterial, anti-inflammatory, antihypertensive, neuroprotective, and antimutagenic effects (14–19). Furthermore, Q extract modulated airway inflammation in an OVA-induced allergic rhinitis model (20). *Saururus chinensis* (SC) is a perennial herb found in China and southern Korea, traditionally used to treat various inflammatory diseases, edema, and jaundice. SC extract showed anti-inflammation, anti-angiogenesis, anti-asthma, and anti-atopic dermatitis activities (21–24). The unique properties of these medicinal plant extracts show that their synergistic use may enhance their immunomodulatory and anti-inflammatory effects. However, the effects of a Q/SC mixture in asthma remain unclear. Therefore, the purpose of this study is to evaluate the therapeutic efficacy of a Q/SC mixture in asthma. In this study, we sought to provide insights that can guide the development of safer and more effective therapies for asthma and related inflammatory disorders by elucidating their combined mechanisms of action. To achieve this goal, OVA-induced allergic asthma models were generated and treated with the Q/SC mixture.

## 2 Materials and methods

### 2.1 Animal models

All trials were conducted using six-week-old female BALB/c mice (Orient Bio Ltd., Seongnam, Korea) housed in a pathogen-free facility. After a 7-day acclimation period in the animal facility, we used a mouse model that showed no physical signs of illness and gained weight normally. To create an allergic asthma mouse model, we mixed 50 μg of ovalbumin (OVA) (A5503, Sigma-Aldrich, St. Louis, MO, United States) and 1.32 mg of aluminum hydroxide (Alum) (Sigma-Aldrich) in 200 μL of phosphate-buffered saline (PBS). The mice were sensitized intraperitoneally twice at 1-week intervals. Two weeks post-sensitization, allergen exposure was administered via intratracheal injection of 50 μg of OVA daily for 7 days. Dexamethasone (DEX, 3 mg/kg) and the Q/SC extract (50, 100, and 200 mg/kg) were orally administered 1 h before the OVA challenge. DEX, a corticosteroid commonly prescribed for treating allergies and other respiratory disorders, served as the positive control. The normal control (NC) group was administered the same amount of PBS. The animals were euthanized 24 h after the final OVA exposure, and samples were collected for study.

### 2.2 Preparation of *Chinese quince* and *Saururus chinensis* extract

Q/SC extract was obtained from Novarex (Cheongju, Chungbuk, Korea). The fruits of Q and aerial parts of SC were used for the Q/SC extract. Ursolic acid and miquelianin were used as indicative compounds, with their quantities in the Q/SC extract analyzed using high-performance liquid chromatography (HPLC) to ensure the quality of the extraction process. C18 column (4.6 × 150 mm, 5 μm) and C18 column (4.6 × 250 mm, 5 μm) were used in HPLC.

### 2.3 Collection and preparation of bronchoalveolar lavage fluid, serum samples, and lung tissues

Bronchoalveolar lavage (BALF) collection and cytostaining from anesthetized mice were conducted as previously reported (25, 26). Following tracheal lavage, the lungs were immediately resected, and the left lung tissues were preserved in 10% (v/v) buffered formalin. A portion of the right lung tissue was placed in RNA later solution (AM7020, Invitrogen, Waltham, MA, United States), while the remaining tissues were stored in a tube at −80°C. Blood samples were obtained from the abdominal vein, and the serum was used for the OVA-specific IgE assay.

### 2.4 Enzyme-linked immunosorbent assay

A mouse IgE enzyme linked immunosorbent assay (ELISA) kit (Thermo Fisher Scientific, Waltham, MA, United States) was used to measure serum OVA-specific IgE levels. ELISA kits (R&D Systems, Minneapolis, MN, United States) were used to quantify the cytokines TNF-*α*, IL-17, and IL-1β in lung homogenates, while an ELISA kit (Cusabio Biotech, Wuhan, China) was used to measure inducible nitric oxide synthase (iNOS) levels. ELISA kits (R&D Systems) were also used to determine IL-4 and IL-13 levels in BALF. All analyses were conducted following the manufacturer’s guidelines.

### 2.5 Real-time quantitative polymerase chain reaction

As previously described, total RNA was extracted from a portion of the right lung tissue sample, and quantitative real-time RT-PCR was conducted (27). Target gene levels were normalized to Glyceraldehyde 3-phosphate dehydrogenase. The primer sequences are listed in Table 1.

**Table 1:** Primer sequences used.

Target gene	Forward primer (5′ to 3′)	Reverse primer (5′ to 3′)
IL-1β	TGGACCTTCCAGGATGAGGACA	GTTCATCTCGGAGCCTGTAGTG
IL-4	CCCCCAGCTAGTTGTCATCC	CCCTTCTCCTGTGACCTCGT
IL-5	TGGAGATTCCCATGAGCACAGT	GCCTCATCGTCTCATTGCTTGT
IL-13	GGAGCTGAGCAACATCACACAA	GCGGCCAGGTCCACACT
TNF-α	CCCAGACCCTCACACTCAGAT	CCTCCACTTGGTGGTTTGCT
IL-17F	TGCTACTGTTGATGTTGGGAC	CAGAAATGCCCTGGTTTTGGT
MUC5AC	TGCATGCGTACCTGCCAGAA	CACACTGCATTGTGCCCTCA
iNOS	AACGGAGAACGTTGGATTTG	CAGCACAAGGGGTTTTCTTC
GAPDH	TGTGTCCGTCGTGGATCTGA	CCTGCTTCACCACCTTCTTGA

IL, Interleukin; TNF, Tumor necrosis factor; GAPDH; Glyceraldehyde 3-phosphate dehydrogenase; iNOS, Inducible nitric oxide synthase.

### 2.6 Lung tissue histology

The left lung tissue, preserved in 10% (v/v) buffered formalin, was stained with hematoxylin and eosin to assess inflammatory cells. The periodic acid-Schiff (PAS) kit (ab150680, Abcam, Cambridge, United Kingdom)was used to stain goblet cells, while the toluidine blue protocol was used to stain the mast cells. Stained slides were analyzed using light microscopy, and the extent of goblet cell hyperplasia and inflammatory cell infiltration was scored using a subjective scale, as previously described (28–30). Mast cells were counted in toluidine blue-stained sections, with at least three regions analyzed per section.

### 2.7 Western blotting

Protein extraction and quantification from lung tissue, followed by WB analysis, were conducted as previously reported (31–33). The primary antibodies used were STAT6 (Cat# 9362, Cell Signaling, Beverly, MA, United States), p-STAT6 (Cat# 56554), and *β*-actin (Cat# 5125s). The detected immune-reactive bands were evaluated using ImageJ software.

### 2.8 Statistical analysis

Statistical analyses were conducted using GraphPad Prism version 10.2.2 (GraphPad Software, San Diego, CA, United States). Differences between multiple experimental groups were assessed using a one-way analysis of variance, with post-hoc Tukey and Dunnett tests conducted afterward. A *p*-value of <0.05 was regarded as statistically significant. Data are shown as the mean ± SEM.

## 3 Results

### 3.1 Effect of Q/SC extract on inflammatory cells infiltration in BALF

**Figure 1:**
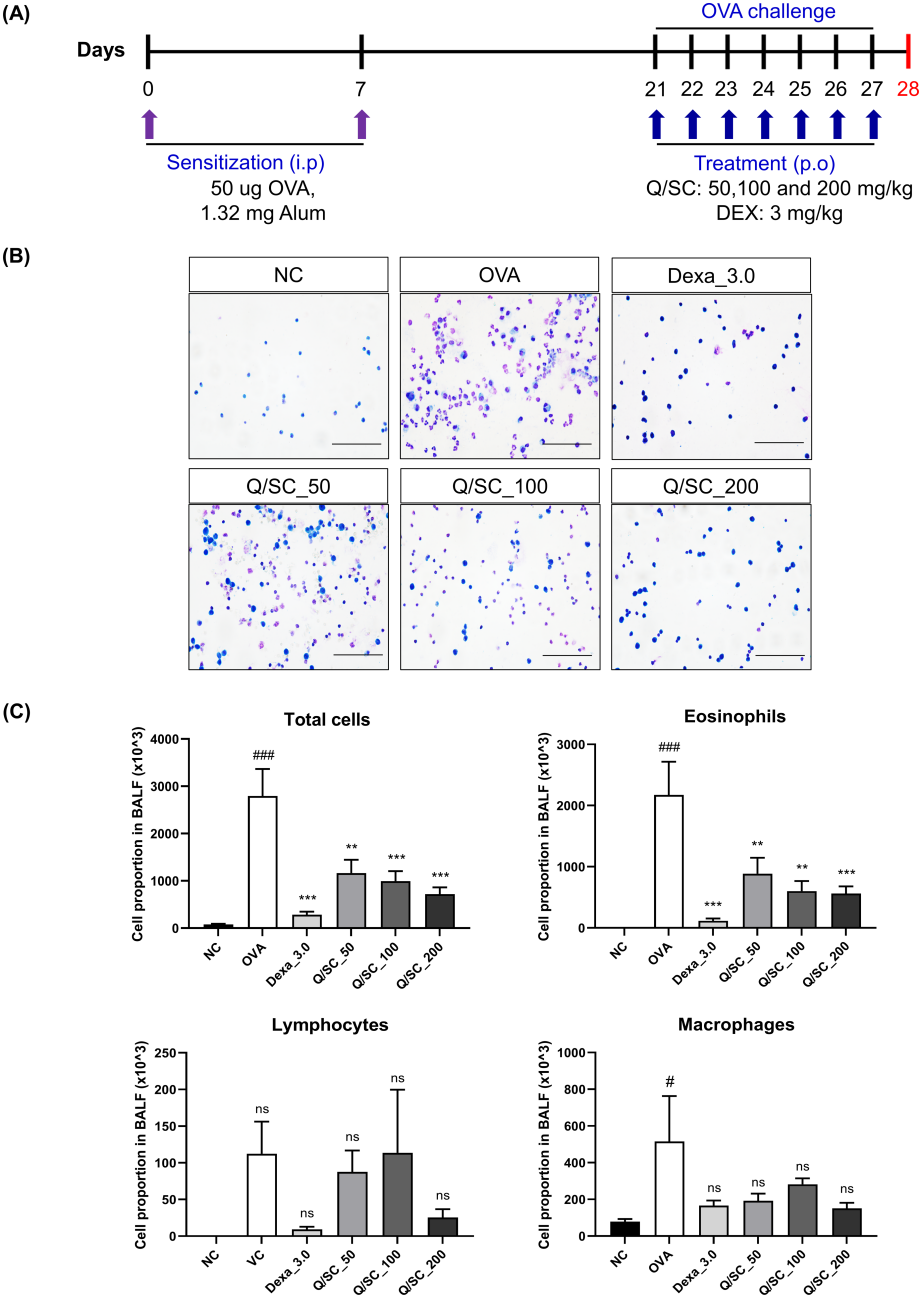
Experimental schedule for the asthma mouse model and the effect of *Chinese quince/Saururus chinensis* (Q/SC) extract on inflammation in bronchoalveolar lavage fluid (BALF). **(A)** Experimental procedure for the allergic asthma model and administration of dexamethasone (DEX) and Q/SC extract. **(B)** BALF cells are plated on clean glass slides and stained with Diff-Quik. Scale bar = 50 μm. **(C)** Total and differential cell counts are conducted using a cell counter under light microscopy. Data are shown as the means ± SEMs. ^###^*p <* 0.001 vs. the normal control group. ^***^p < 0.001 and ^**^p < 0.01 vs. the ovalbumin-challenged group. NC, normal control; OVA, ovalbumin; DEX, dexamethasone; Q, *Chinese quince*; SC, *Saururus chinensis*; BALF, bronchoalveolar lavage fluid.

To determine the anti-inflammatory effect of Q/SC extract on allergic asthma, an allergic asthma mouse model was established. Cell infiltration in the BALF was more pronounced in the OVA-challenged group than it was in the NC group. The number of inflammatory cells, particularly eosinophils, was markedly higher in the OVA-challenged group. Conversely, the eosinophil counts were notably lower in the DEX and Q/SC groups. Furthermore, the total cell count and number of inflammatory cells, including lymphocytes and eosinophils, were notably higher in the OVA-challenged group than it was in the NC group. Meanwhile, compared with the OVA-challenged group, the Q/SC group had notably lower total cell count and number of eosinophils (Figures 1A–C).

### 3.2 Effect of Q/SC on inflammatory cell recruitment and IgE production

**Figure 2:**
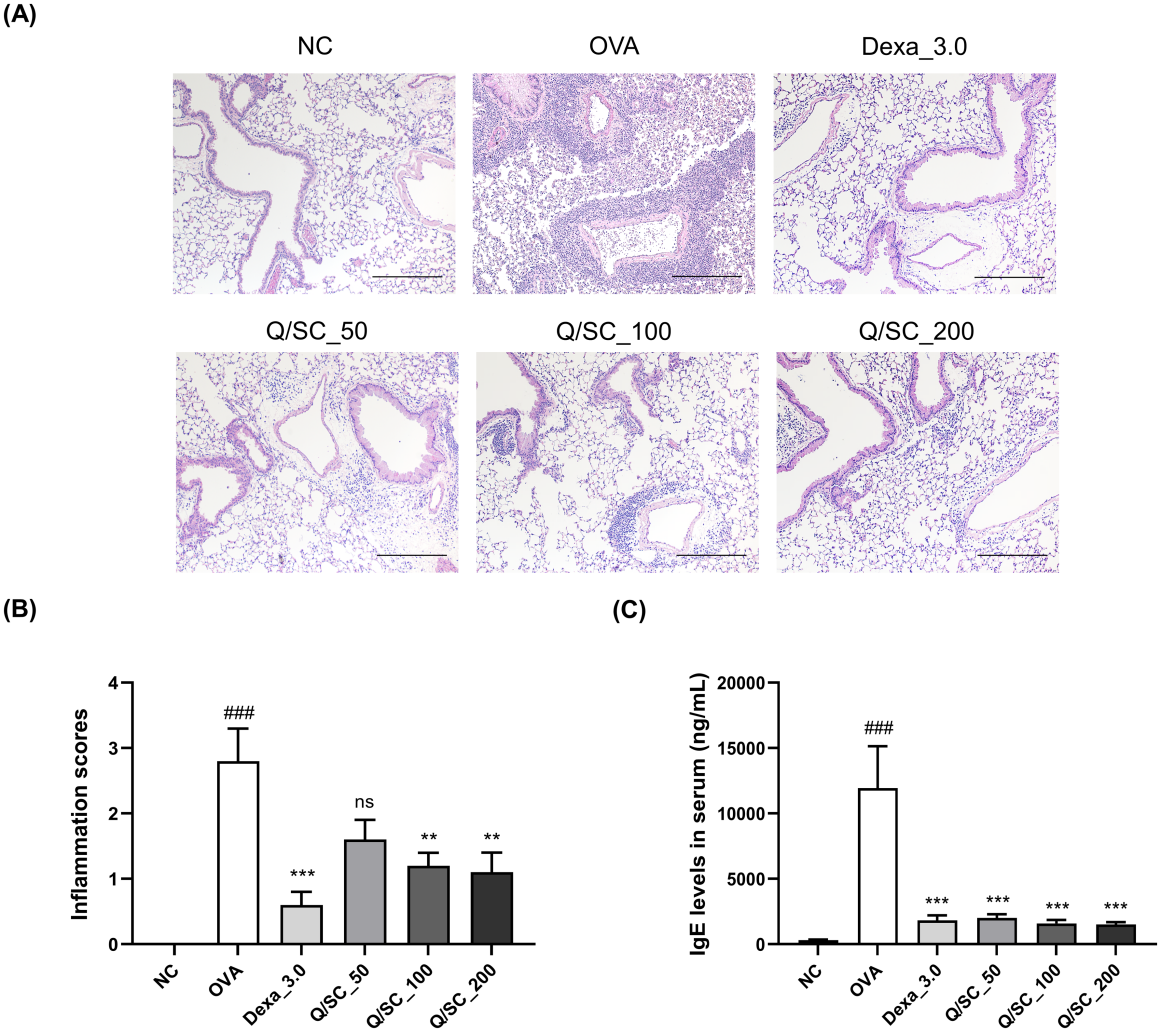
Effects of *Chinese quince/Saururus chinensis* (Q/SC) extract on immunoglobulin E (IgE) production and immune cell infiltration in ovalbumin (OVA)-induced allergic asthma mice. **(A)** The paraffin-embedded lung sections were stained with hematoxylin and eosin. Scale bar = 200 μm. **(B)** Lung inflammatory scores are determined using histological analysis of lung tissues. **(C)** Serum IgE levels are detected using enzyme-linked immunosorbent assay. Data are displayed as the means ± SEMs. ^###^*p* < 0.001 vs. normal control group. ^***^p < 0.001 and ^**^p < 0.01 vs. OVA-challenged group. NC, normal control; OVA, ovalbumin; IgE, immunoglobulin E.

The effect of Q/SC extract on airway inflammation was evaluated via tissue staining. Consequently, inflammatory cell infiltration was increased in the OVA-challenged group, whereas it was significantly reduced in the DEX, Q/SC-100, and Q/SC-200 treatment groups (Figures 2A,B). IgE is secreted from B cells activated by antigens and causes atopic dermatitis, allergic rhinitis, and asthma (12). To examine the influence of the Q/SC extract on IgE production, IgE serum levels were measured. Consequently, IgE levels were increased in the OVA-challenged group, whereas they were decreased in all Q/SC treatment groups (Figure 2C). The findings show that the Q/SC extract, particularly at doses of 100 mg/kg and 200 mg/kg, attenuated OVA-induced allergic asthma.

### 3.3 Effect of Q/SC extract on airway inflammation

**Figure 3:**
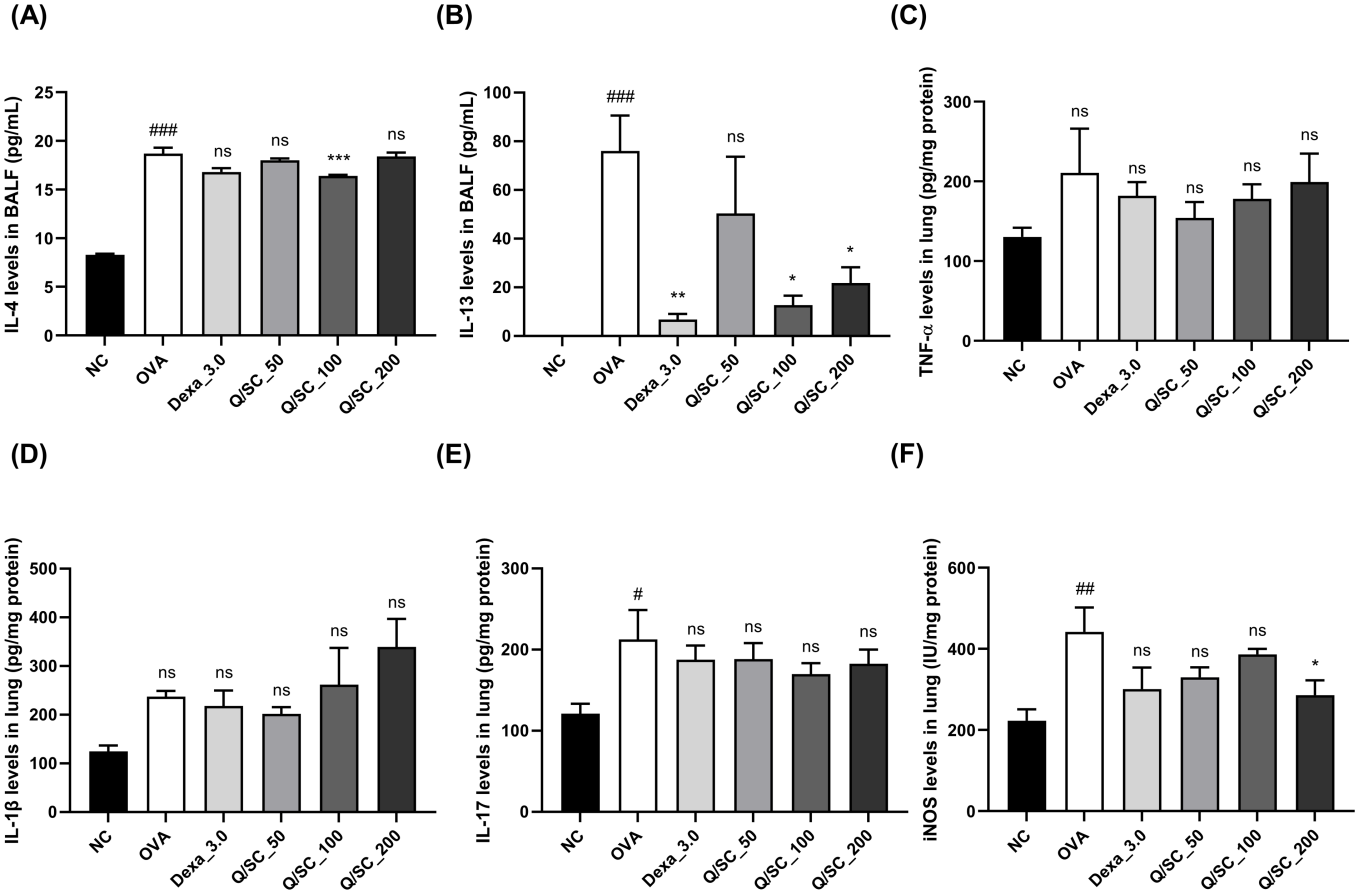
Effects of *Chinese quince/Saururus chinensis* (Q/SC) extract on non-type 2 and type 2 inflammation and inducible nitric oxide synthase (iNOS) expression in ovalbumin (OVA)-induced allergic asthma. **(A,B)** Enzyme-linked immunosorbent assay (ELISA) is conducted to detect the levels of interleukin (IL)-4 and IL-13, as type 2 cytokine levels in bronchoalveolar lavage fluid (BALF). **(C–F)** ELISA is also conducted to measure the levels of interleukin (IL)-1β, Tumor necrosis factor (TNF)-*α*, and IL-17, as non-type 2 cytokines, and iNOS expression in lung homogenates. Data are displayed as the means ± SEMs. ^###^*p* < 0.001, ^##^*p* < 0.01, and ^#^*p* < 0.05 vs. the normal control group. ^***^p < 0.001, ^**^p < 0.01, and ^*^p < 0.05 vs. the OVA-challenged group. iNos, inducible nitric oxide synthase; OVA, ovalbumin; BALF, Bronchoalveolar lavage fluid; ELISA, Enzyme-linked immunosorbent assay.

**Figure 4:**
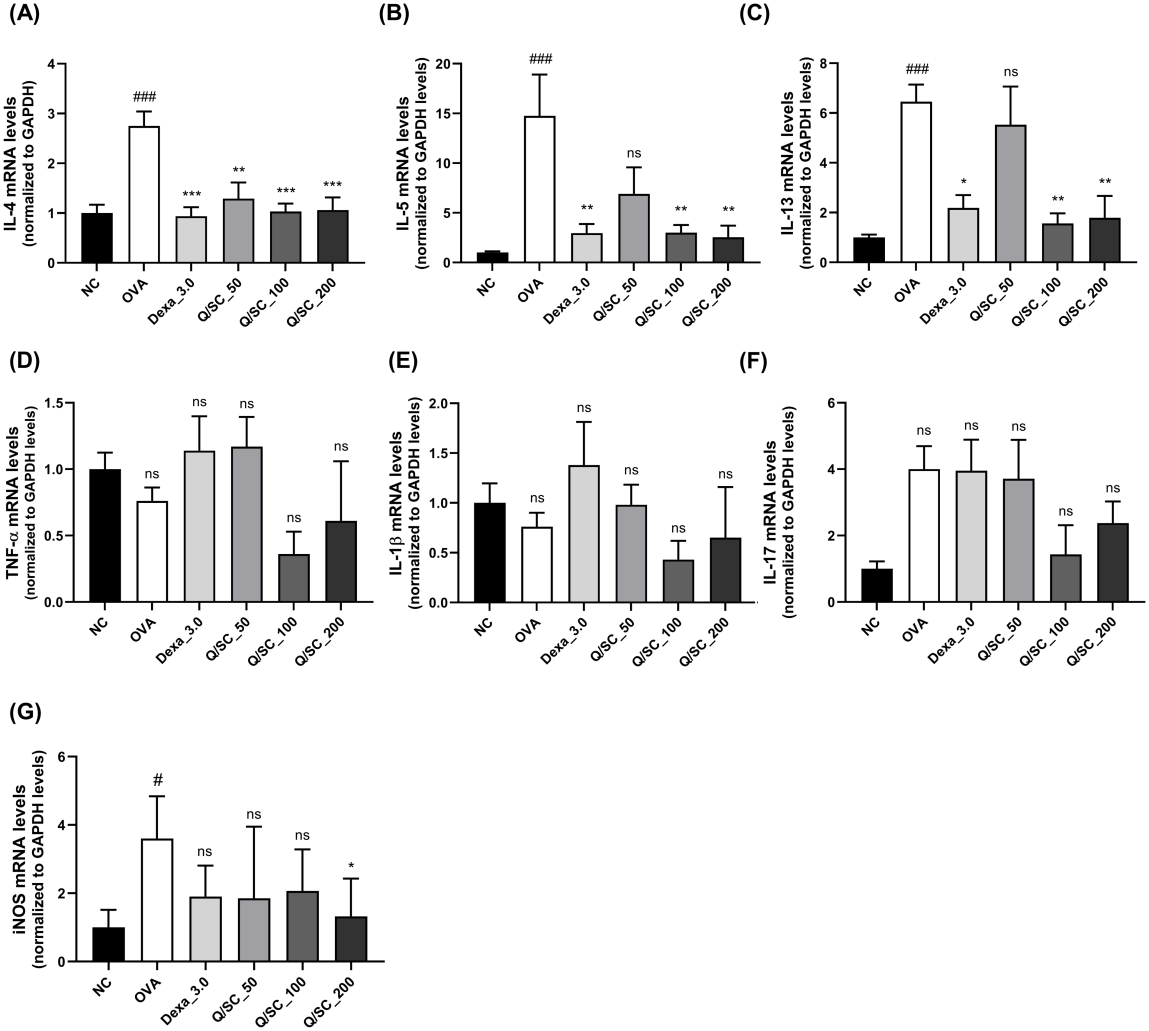
*Chinese quince/Saururus chinensis* (Q/SC) extract suppresses mRNA expressions of type 2-related cytokines and inducible nitric oxide synthase (iNOS). **(A–C)** The mRNA levels of type 2 cytokines (interleukin (IL)-4, IL-5, and IL-13), **(D–F)** non-type 2 cytokines (IL-1β, TNF-α, and IL-17), and **(G)** iNOS in homogenates are assessed using reverse transcription quantitative polymerase chain reaction and are normalized to glyceraldehyde 3-phosphate dehydrogenase levels. Data are displayed as the means ±SEM. ^###^*p* < 0.001 and ^#^*p* < 0.05 vs. the normal control (NC) group. ^***^p < 0.001, ^**^p < 0.01, and ^*^p < 0.05 vs. the ovalbumin-challenged group.

Allergic asthma is primarily associated with type 2 immune responses, which are modulated by type 2 cytokines that induce pathological changes at the site of inflammation and exacerbate asthma symptoms (34). Therefore, to determine the effectiveness of Q/SC extract on allergic airway inflammation, the levels of type 2 and non-type 2 inflammatory cytokines in BALF and lung homogenates were measured using an ELISA kit. Compared with the NC group, the OVA-challenged group showed higher levels of type 2 and non-type 2 cytokines in BALF and lung homogenates. In BALF, IL-4 levels were decreased in the Q/SC-100 treatment group, and IL-13 levels were notably decreased in the Q/SC-100 and Q/SC-200 treatment groups (Figures 3A,B). Additionally, non-type 2 cytokines levels in lung homogenates were not reduced in the Q/SC treatment groups (Figures 3C–E). Increased iNOS expression is common in allergic airway inflammation; however, under normal conditions, iNOS is either absent or present in minimal amounts in most cell types and tissues. iNOS activity can be upregulated by various inflammatory factors, including allergen exposure, leading to bronchial hyperresponsiveness and contributing to eosinophil recruitment (35–37). In this study, iNOS production in lung homogenates was significantly lower in the Q/SC-200 treatment group than it was in the OVA-challenged group (Figure 3F). Furthermore, the effect of Q/SC extract was investigated by assessing mRNA expression levels for type 2 and non-type 2 mediated allergic responses in lung homogenates. The amount of type 2 cytokines, but not that of non-type 2 cytokines, was significantly reduced in the Q/SC-100 and Q/SC-200 treatment groups (Figures 4A–F). Like the ELISA results, the mRNA expression levels showed that iNOS expression was significantly decreased in the Q/SC-200 treatment group (Figure 4G). These findings show that Q/SC-100 and Q/SC-200 treatment alleviated airway inflammation by regulating type 2-related cytokines and iNOS production.

### 3.4 Effect of Q/SC extract on OVA-induced mucus production and the STAT6 signaling pathway

**Figure 5:**
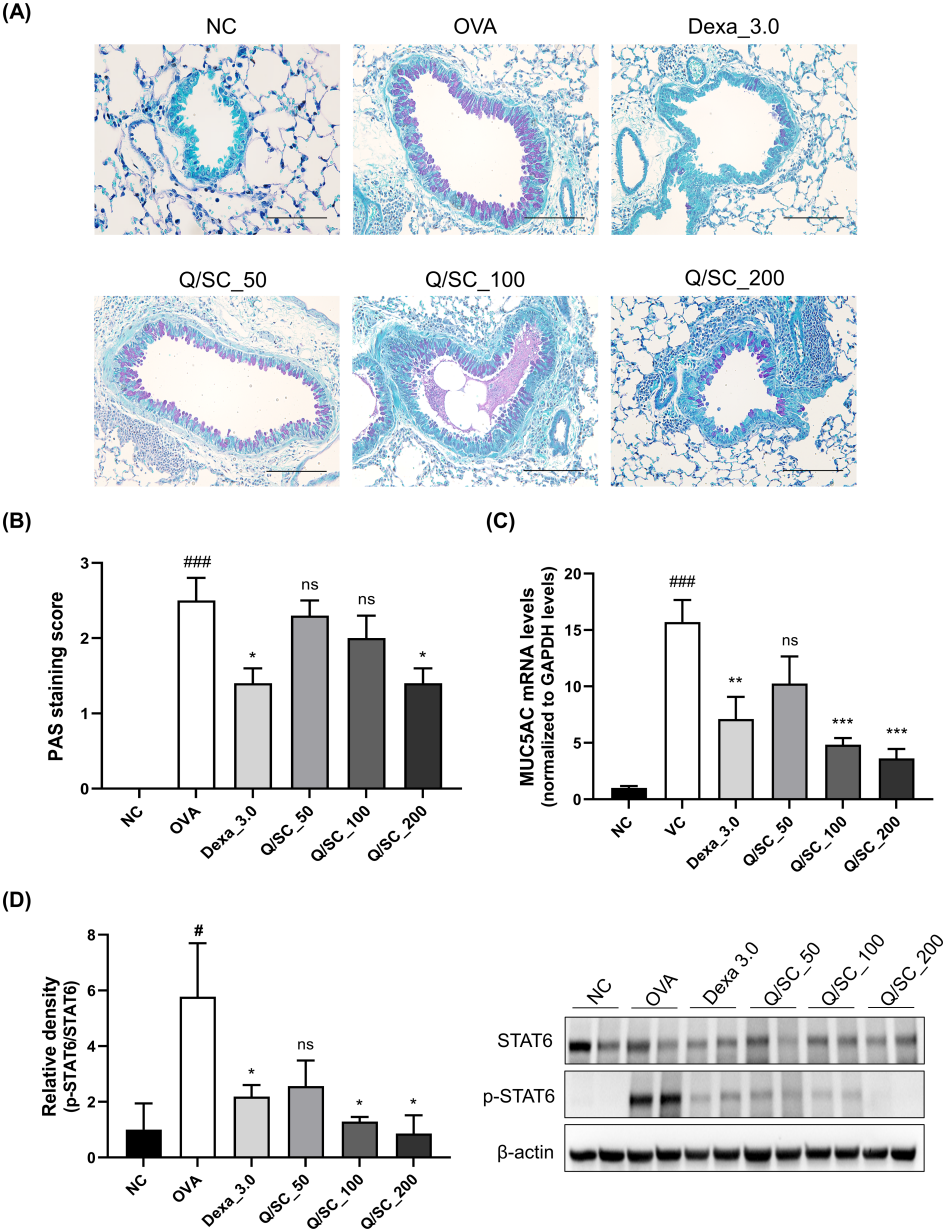
Effect of the *Chinese quince/Saururus chinensis* (Q/SC) extract on mucus production and the STAT6 signaling pathway in ovalbumin (OVA)-induced allergic asthma. **(A)** Periodic acid-Schiff staining is used to assess goblet cell hyperplasia in the epithelium. Scale bar = 100 μm. **(B)** Quantification of goblet cells in lung tissues. **(C)** The mRNA level of the mucus gene MUC5AC is quantified using reverse transcription quantitative polymerase chain reaction and is normalized to glyceraldehyde 3-phosphate dehydrogenase levels. **(D)** STAT6 phosphorylation is measured using western blot. The total forms of each protein are used as loading controls. Data are displayed as the means ± SEMs. ^###^*p* < 0.001 vs. the normal control group. ^***^p < 0.001, ^**^p < 0.01, and ^*^p < 0.05 vs. the OVA-challenged group.

Goblet cell hyperplasia and excessive mucus production in the bronchi are the most common features observed in allergic asthma models. Therefore, PAS staining was carried out to evaluate the effect of the Q/SC extract on mucus production. The results showed higher mucus production in the lung epithelium of the OVA-challenged group than in that of the NC group. Conversely, mucus production was significantly lower in the DEX and Q/SC-200 treatment groups than in that of the OVA-challenged group (Figures 5A,B). Furthermore, MUC5AC mRNA expression was significantly higher in the OVA-challenged group than it was in the NC group, whereas it was significantly lower in the DEX, Q/SC-100, and Q/SC-200 treatment groups (Figure 5C). Finally, STAT6 phosphorylation was markedly increased in the OVA-challenged group, whereas it was substantially decreased in the DEX and Q/SC treatment groups (Supplementary Figure S1). STAT6 phosphorylation was eliminated in the Q/SC-200 treatment group (Figure 5D). Collectively, these results show that high concentrations of Q/SC extract inhibit MUC5AC expression by suppressing the STAT6 signaling pathway.

## 4 Discussion

In this study, we found that high concentrations of the Q/SC extract significantly inhibited serum IgE production, type 2 cytokine levels, and iNOS expression. Additionally, high concentrations of the Q/SC extract effectively inhibited mucus secretion and immune cell infiltration in the lung tissues of OVA-challenged mice with allergic airway inflammation, similar to the effect of DEX. Collectively, our results show that high concentrations of the Q/SC extract are effective against allergen-induced airway inflammation in asthma.

Asthma is a chronic respiratory condition characterized by reversible airway obstruction and bronchial hyperresponsiveness. It affects approximately 300 million people worldwide (38). In most patients, inhaled corticosteroids and long-acting β2 agonists are used to control asthma symptoms; however, long-term and high-dose use of these drugs can lead to side effects (39). Additionally, some patients require repeated use of systemic steroids because of poor response to drug treatment, leading to steroid-related side effects. Therefore, there is increasing awareness of the significance of medicinal plants for the effective and safe management of asthma symptoms (40). Q extract may be effective against allergic inflammation, while SC extract may modulate lung inflammatory diseases in allergic asthma (41). However, the potential therapeutic mechanisms of a Q/SC mixture in allergic asthma-associated lung inflammation are not well understood. Asthma is categorized into type 2 and non-type 2 endotypes, depending on the immune cell types and inflammation patterns. Allergic asthma is classified as a type 2 endotype marked by increased eosinophilic inflammation and expression of type 2 cytokines, including IL-4, IL-5, and IL-13 (42). IL-4 promotes IgE synthesis from B cells and Th2 cell differentiation, whereas IL-5 is involved in recruiting eosinophils to inflammatory sites. IL-13 is essential in excessive mucus secretion, immune cell influx, and airway hyperresponsiveness (43, 44).

Nitric oxide (NO) plays an important role as an endogenous regulator of airway and distal lung constriction. NO levels are accordingly used as an indicator of eosinophilic airway inflammation. iNOS, the enzyme that produces NO, has increased transcriptional expression owing to the inflammatory cytokines IL-4 and IL-13 and is directly involved in eosinophil recruitment. Moreover, iNOS expression is associated with inflammation of the upper and lower airways (45–47). Therefore, regulating IgE, iNOS, and type 2 cytokines is crucial for improving allergic asthma. However, the expression of non-type 2 cytokines was not suppressed in the DEX group in this study, which aligns with reports that non-type 2 asthma was not controlled by DEX, a corticosteroid (48, 49). Airway mucus hypersecretion leads to a higher number of goblet cells in the airway epithelium, and this, in turn, leads to elevated MUC5AC expression, exacerbating asthma. MUC5AC expression is higher in patients with asthma than in healthy individuals. Additionally, the transcript levels of MUC5AC are significantly elevated during allergen-induced airway inflammation, whereas those of MUC1, MUC2, MUC3, MUC4, MUC5B, and MUC13 remain unchanged in the mouse lung tissue (50–54). Furthermore, MUC5AC levels are higher in type 2 asthma than in non-type 2 asthma (55, 56). In mouse lungs, IL-13 upregulates MUC5AC through a STAT6-dependent pathway, where STAT6 plays a critical function in regulating the transcription of several IL-4/IL-13-dependent genes. In previous studies, siRNA knockdown of STAT6 significantly reduced IL-4/IL-13-induced MUC5AC promoter activity (54, 57). In this study, goblet cell and MUC5AC expressions and STAT6 pathway phosphorylation were markedly elevated in OVA-challenged mice than it was in NC mice. Importantly, treatment with high concentrations of the Q/SC extract markedly reduced goblet cell and MUC5AC expressions and STAT6 pathway phosphorylation. This finding shows that high concentrations of the Q/SC extract have therapeutic potential for inhibiting mucus production and secretion in allergic airway inflammation. However, this study has some limitations in elucidating the molecular mechanisms through which Q/SC extract is involved in asthma. Future studies should focus on using asthma models to clarify the mechanism of action of Q/SC extract with a more rigorous research design.

## 5 Conclusion

High concentrations of the Q/SC extract effectively alleviate allergic airway inflammation by suppressing OVA-induced eosinophilic airway inflammation and type 2 cytokine secretion. Additionally, this extract effectively inhibits the STAT6 signaling pathway and reduces mucus hyperproduction. Thus, high concentrations of the Q/SC extract have potential as a treatment for managing allergic airway inflammatory diseases, including asthma.

## Data Availability

The original contributions presented in the study are included in the article/Supplementary material, further inquiries can be directed to the corresponding authors.
