# Supplementary figures and images for: Therapeutic effects of a combination of Chinese quince and Saururus chinensis extract on allergic airway inflammation in an ovalbumin-induced asthma mouse model
Source: Front Nutr. 2025 Jul 10;12:1613413. doi: 10.3389/fnut.2025.1613413 (PMC12286951; doi:10.3389/fnut.2025.1613413)

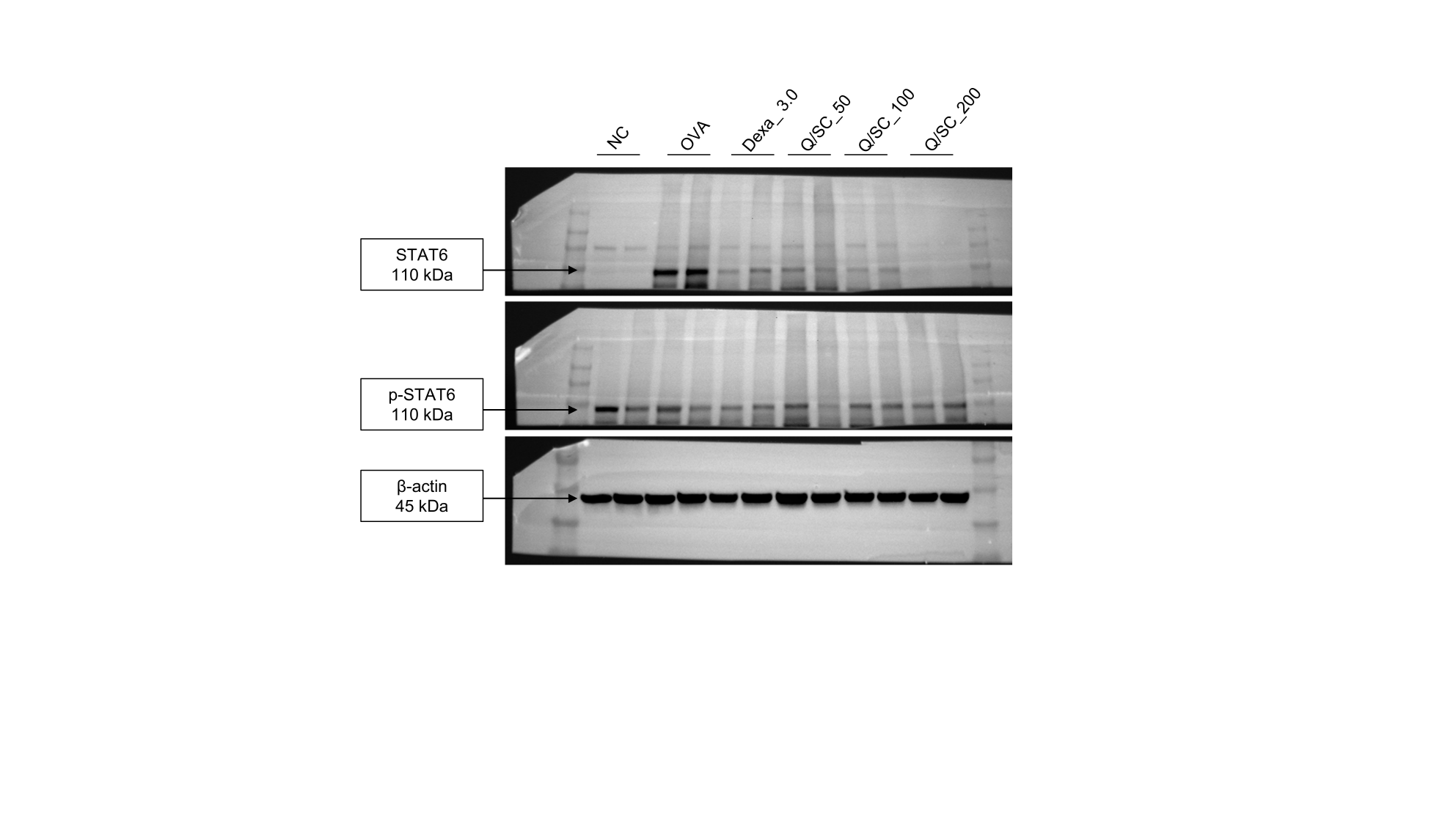

Supplement: Supplementary file 1 [file Image_1.tif]
